# Quantitative Genetics of Feed-Efficiency-Related Traits for the Pacific Whiteleg Shrimp *Penaeus vannamei* in a Plant-Based Diet Environment

**DOI:** 10.3390/biology13121012

**Published:** 2024-12-04

**Authors:** Ping Dai, Xiangyan Zhu, Sheng Luan, Juan Sui, Xianhong Meng, Jiawang Cao, Jian Tan, Jie Kong

**Affiliations:** 1 State Key Laboratory of Mariculture Biobreeding and Sustainable Goods, Yellow Sea Fisheries Research Institute, Chinese Academy of Fishery Science, Qingdao 266071, China; daiping@ysfri.ac.cn (P.D.); luansheng@ysfri.ac.cn (S.L.); suijuan0313@126.com (J.S.); caojw@ysfri.ac.cn (J.C.); tannjian@163.com (J.T.); kongjie@ysfri.ac.cn (J.K.); 2 Laboratory for Marine Fisheries Science and Food Production Processes, Qingdao Marine Science and Technology Center, Qingdao 266237, China; 3 Fisheries College, Zhejiang Ocean University, Zhoushan 316022, China; zhuxiangyan2021@126.com; 4 BLUP Aquabreed Co., Ltd., Weifang 261311, China

**Keywords:** *Penaeus vannamei*, fishmeal, plant-based diet, feed efficiency, residual feed intake, genetic parameter

## Abstract

Selecting shrimp strains that perform well on a plant-based diet holds promise for solving the fishmeal (FM) shortage issue, with genetic improvement of feed efficiency (FE) being a key focus. This study compared a plant-based (fishmeal-free) diet with a 25% FM diet for the Pacific whiteleg shrimp (*Penaeus vannamei*) to determine whether the FE-related traits, including residual feed intake (RFI), average daily weight gain (ADG), and daily feed intake (DFI), exhibit diet-specific genetic variations and genotype-by-diet (genotype × diet) interactions. Results showed that RFI was significantly higher under the plant-based diet treatment compared to the FM-based diet treatment (0.0049 g/d vs. –0.0045 g/d, *p* < 0.001), while ADG was notably lower (0.043 g/d vs. 0.110 g/d, *p* < 0.001), indicating that a plant-based diet may lead to reduced yields and impact the profitability of shrimp culture. In the case of being unable to estimate common environmental effects, the heritability estimates of RFI under the plant-based and FM-based diets were 0.743 ± 0.157 and 0.440 ± 0.125, respectively, and those of ADG and DFI ranged from 0.314 ± 0.121 to 0.947 ± 0.158. Genetic correlations between diets for RFI and DFI were 0.646 ± 0.162 and 0.549 ± 0.163, respectively, suggesting a degree of genotype × diet interactions. In contrast, the genetic correlation between diets for ADG was only 0.296 ± 0.259, indicating a strong genotype × diet interaction. These findings highlight the importance of considering selective breeding for improved feed efficiency in a plant-based diet environment.

## Simple Summary

Selecting shrimp strains for optimal performance on a plant-based diet holds promise for solving the fishmeal (FM) shortage issue, with genetic improvement of feed efficiency (FE) being a key focus. This study compared a plant-based (fishmeal-free) diet with a 25% fishmeal diet for *Penaeus vannamei*, focusing on feed efficiency traits like residual feed intake (RFI) and average daily weight gain (ADG). Results showed higher RFI (0.0049 g/d vs. −0.0045 g/d) and lower ADG (0.043 g/d vs. 0.110 g/d) on the plant-based diet, indicating potential yield reductions. Heritability estimates for RFI were 0.743 ± 0.157 (plant-based diet) and 0.440 ± 0.125 (fishmeal-based diet). Genetic correlations between both diets for RFI (0.646 ± 0.162) and ADG (0.296 ± 0.259) suggested different degrees of genotype × diet interactions, underscoring the need for selective breeding in plant-based diets.

## 1. Introduction

The Pacific whiteleg shrimp (*Penaeus vannamei*) has seen a significant increase in global production, expanding from 154,500 tonnes in 2000 to 5.8 million tonnes in 2020 [1]. It was the top species produced in that year, closely followed by grass carp (*Ctenopharyngodon idellus*) and cupped oysters (*Crassostrea* spp.) [1]. The growth of its industry relies heavily on the development of more cost-effective feeds, as feed costs account for 40 to 60% of the total expenses in shrimp production [2]. Protein is the most expensive component in the feed for farmed whiteleg shrimp [3]. Fishmeal (FM) is a highly preferred protein feedstuff due to its essential nutrients like amino acids, fatty acids, cholesterol, vitamins, attractants, and growth factors [4]. It is usually the primary protein source of commercial feed formulations for shrimp [5], and also a major component in the diets of farmed fish species [6]. However, global supplies of FM have reached a plateau, yet demand continues to increase, especially in aquatic feeds, leading to higher costs and limited availability [7].

Replacing FM with plant protein feedstuffs like soybean meal (SBM) and peanut meal (PM) is an economically beneficial solution due to their cost-effectiveness and consistent nutrient supply [8,9,10]. Complete replacement of FM with plant protein sources in feed for *P. vannamei* has been achieved. For example, Amaya et al. [5] demonstrated that fishmeal can be completely replaced using a combination of soybean and corn gluten in shrimp feeds without compromising production and economic performance of *P. vannamei* reared in ponds. Bauer et al. [11] found that fishmeal can be completely replaced with a mixture of soy protein concentrate and microbial floc meal in diets, without adverse effects for *P. vannamei* juveniles. 

In addition to developing scientifically formulated nutritional strategies, selecting strains that exhibit good production performance on plant-based (fishmeal-free) diets holds promise for addressing the shortage of fishmeal (FM). Although research on shrimp in this context is limited, studies on fish have identified genotypes capable of efficiently utilizing plant-based diets [12,13,14,15,16]. For instance, rainbow trout bred for four generations on a fishmeal-free diet showed comparable growth to those fed a 34% FM diet [17]. Therefore, selecting strains that are tolerant to plant-based diets could be a viable approach. However, Yamamoto et al. [18] found that Amago salmon *Oncorhynchus masou ishikawae* selectively bred for growth on a low-FM (5%) diet exhibit a good response to the low-FM diet, largely due to an increased feed intake with a particular preference for the diet. To avoid situations where significant growth is attributed to excessive feed intake, resulting in poor feed utilization efficiency (FE), it is essential to prioritize FE traits alongside growth traits in shrimp breeding. Emphasizing high FE can help minimize feed usage and lower production costs effectively. 

A key factor in selective breeding for feed efficiency (FE) is determining which traits to include in the selection index. Since any genetic changes observed in ratio traits such as feed efficiency ratio (FER) and feed conversion ratio (FCR) cannot be directly attributed to the genetic variation in growth or feed intake, or their combination, selecting animals based solely on FER or FCR will result in poor selection response. Residual feed intake (RFI), defined as the difference between the actual amount of feed consumed and the expected feed consumption estimated by a regression model which takes into account the feed requirements for maintenance and growth as independent variables, is considered a better measure of FE [19,20]. A negative RFI means an animal consumes less feed than expected, while a positive RFI indicates higher feed intake. Animals with low RFI are more efficient, and those with high RFI are less efficient. RFI has been widely used in the study of feed efficiency in livestock and poultry [21,22,23]. Research on RFI in aquatic animals is still relatively limited, with most focusing on fish, including African catfish *Clarias gariepinus* [24], rainbow trout *O. mykiss* [25], and Nile tilapia *Oreochromis niloticus* [26]. Additionally, there have been a few reports on RFI in whiteleg shrimp *P. vannamei* [19], oriental river prawn *Macrobrachium nipponense* [27], and Pacific abalone *Haliotis discus hannai* [28].

Genetic improvement of FE traits is crucial for selecting shrimp strains that perform well on a plant-based diet. Dai et al. [19] reported high heritability estimates for FE traits, including FER and RFI, in *P. vannamei* when fed a FM-based diet. However, limited information is available about the FE of *P. vannamei* fed a plant-based diet, especially whether it has the potential for selective breeding. In addition, it remains uncertain whether there is a genotype-by-diet (genotype × diet) interaction effect for FE between plant-based and FM-based diets. The future use of plant-based diets could impact breeding programs if genotype × diet interaction occurs. Aiming to explore how the utilization of a plant-based diet impacts the genetic improvement of FE in *P. vannamei*, the present study set up both a plant-based diet (fishmeal-free) and a FM-based diet (25% FM) environment to determine whether RFI and the related traits, including average daily weight gain (ADG) and daily feed intake (DFI), exhibit diet-specific phenotypic and genetic variations, and to estimate the genotype × diet interactions for these traits.

## 2. Materials and Methods

### 2.1. Shrimp Production

In 2019, a base population was established using three commercial strains [29]. The breeding program involved producing discrete generations annually through the construction of many full-sib families which included some half-sib families, following the procedure outlined by Li et al. [30]. The primary target traits were disease resistance and growth. Thirty full-sib families created in 2023, including four maternal half-sib families, were selected for an experiment. The shrimp reproduction and culture were carried out at the whiteleg shrimp breeding center of BLUP Aquabreed Co. Ltd., situated in Weifang, Shandong province, China.

### 2.2. Experimental Design

A plant-based (fishmeal-free) diet and an FM-based diet (25% FM) were utilized in a feeding trial. The formulated pellet diets were prepared according to the methods outlined by Xu et al. [31]. The ingredients and proximate composition of the two diets are presented in Table 1.

When the majority of the shrimp reached approximately 5 cm in size, 30 individuals were randomly selected from each family and evenly split into two groups to receive different diets (*n* = 15 individuals per group). A total of 900 individuals from 30 families were involved in the feeding trial. The trial was conducted in three aquatic housing systems that operated with recirculating water. Each system consisted of 300 independent small tanks with dimensions of 200 mm × 150 mm × 100 mm. The bottom of each tank featured a special mesh designed to allow the drainage of feces while preventing unconsumed feed from escaping. A randomized complete block design was used for assigning shrimp from each diet group of each family to three housing systems. Specifically, 15 shrimp from each diet group of each family were randomly divided into three systems, with 5 individuals placed in each system, and each shrimp was housed separately in one tank.

### 2.3. Feeding Trial

The feeding trial took place at the whiteleg shrimp breeding center of BLUP Aquabreed Co. Ltd. Upon being stocked in independent culture tanks, all shrimp underwent a one-week acclimation period during which they were fed with the FM-based diet. Any dead shrimp were replaced during this acclimation phase. During the trial, the shrimp were fed twice daily at 8:00 and 16:00, with each individual’s pellet diet stored in a separate container. The amount of diet per meal was carefully controlled to ensure consumption within half an hour. Any unconsumed pellet diet was collected in a separate container half an hour after each feeding and then dried until its weight stabilized. Additionally, 80% of the seawater in the tanks was exchanged daily, and the temperature was maintained at 27 ± 1 °C (mean ± SD). Dead shrimp and molts were removed on a daily basis.

The trial lasted for 42 days, during which the body weight of each shrimp was recorded at the beginning and end of the trial, defined as initial body weight (IBW) and final body weight (FBW), respectively. The weight gain (WG) for the entire trial was calculated. For each shrimp, the uneaten diet was collected daily into a container and accumulated until the end of the trial. This weight was equivalent to the amount of diet unconsumed during the trial. The total amount of diet offered during this period was equal to the difference in weight of the diet container at the start and end of the trial. Feed intake (FI) was calculated as the difference between the amount of diet offered and the unconsumed amount. ADG and DFI were calculated as WG and FI divided by 42 days, respectively. The survival rate under each diet was calculated by dividing the number of live individuals at the end of the experiment by 450. Since most of the shrimp could not be sexed at the end of the experiment, sexual information was not collected. 

### 2.4. Statistical Analysis

Referring to the studies of laying hens [32,33], a nonlinear approximation model was utilized to relate feed intake to expected maintenance requirements, with the exponent being estimated. During the growth stage of shrimp, energy intake from feed is primarily used for growth and maintenance. An expected DFI was estimated using a multiple regression model as follows:

(1) $$DFI=b_{1}\times {MW}^{b_{2}}+b_{3}\times ADG$$

where MW is the mid-weight (MW = 1/2(IBW+FBW)), ${MW}^{b_{2}}$ is the metabolic mid-weight, DFI and ADG are described as above, and *b*_1_, *b*_2_, and *b*_3_ are partial regression coefficients. The coefficients were computed with the observed DFI serving as the dependent variable, and the nonlinear regression procedure of nls in R version 4.2.3 was utilized. RFI is equal to the difference between the observed and expected DFI: RFI = DFI_observed_ − DFI_expected_. RFI was determined for each diet treatment and across two diet treatments, respectively. The former was employed for subsequent analysis, while the latter was solely used for comparing between diets.

The normality of the distributions for the three traits (RFI, ADG, and DFI) was assessed using the Kolmogorov–Smirnov test. The statistical significance of differences in traits between diets was evaluated using a two-tailed *t*-test. Variance components for these traits were estimated through bivariate analysis using ASReml 4.1 [34]. Animal models were used to examine phenotypic and genetic correlations between traits under different diets, in which traits recorded under the two diet treatments were treated as distinct traits. Each trait observation under a specific diet treatment was assumed to have an independent variance. A four-generation pedigree was included in the analysis. The model was formulated as follows:

(2) $$y_{ij}=\mu +{Tank}_{i}+b\times {IBW}_{j}+a_{j}+e_{ij}$$

where $y_{ij}$ is the observed value of the *i*th individual; μ is the overall mean; ${Tank}_{i}$ is the fixed effect of the *i*th culture system; ${IBW}_{j}$ is the covariate of initial body weight of the *i*th individual; *b* is the regression coefficient; $a_{j}$ is the random additive genetic effect of the *i*th individual, $a\sim (0,{A\sigma }_{a}^{2}),$ where *A* is the additive genetic relationship matrix among all individuals; $e_{ij}$ is the random residual effect of the *i*th individual, $e\sim \left(0,{I\sigma }_{e}^{2}\right)$. 

The phenotypic variance ($\sigma_{p}^{2}$) was computed as the sum of additive genetic variance ($\sigma_{a}^{2}$) and error variance ($\sigma_{e}^{2}$). Heritability was then determined by the ratio of $\sigma_{a}^{2}$ to $\sigma_{p}^{2}$. Genotype × diet interactions for these traits were assessed based on the estimates of genetic correlation, as described by Ponzoni et al. [35]. 

Phenotypic and genetic correlations between traits for each diet treatment were evaluated by bivariate analysis, utilizing the same animal models as previously described. The inclusion of the full-sib family effect (common environmental effect) in the animal models led to non-convergence or a heritability estimate close to zero; thus, this effect was excluded from the current study. Z-scores were employed to test the significance of differences in genetic parameters between diet treatments or between traits. Additionally, they were utilized to determine whether the estimates significantly deviated from zero or one, as detailed by Nguyen et al. [36].

## 3. Results

### 3.1. Residual Feed Intake

The final dataset utilized for RFI analysis consisted of 391 individuals under the plant-based diet treatment (survival rate = 86.89%) and 425 individuals under the FM-based diet treatment (survival rate = 94.44%). Note that the majority of shrimp deaths in the experiment were caused by accidental jumping out of the rearing tanks.

For the plant-based diet, the model to estimate expected DFI was:

$$DFI=0.125\times {MW}^{0.238}+0.042\times ADG$$

while, for the FM-based diet, the model was:

$$DFI=0.096\times {MW}^{0.313}+0.068\times ADG$$

The regression coefficients in these models varied slightly between the two diets. The determination coefficients (R^2^) for the plant-based and FM-based diets were 0.429 and 0.587, respectively.

In the combined analysis across both diets, the model for expected DFI was:

$$DFI=0.127\times {MW}^{0.231}-0.105\times ADG$$

The R^2^ for this combined model was 0.393. The coefficients *b*_1_ and *b*_2_ in this model resembled those in the plant-based diet model, but *b*_3_ differed notably from those in both individual diet models. 

**Figure 1:**
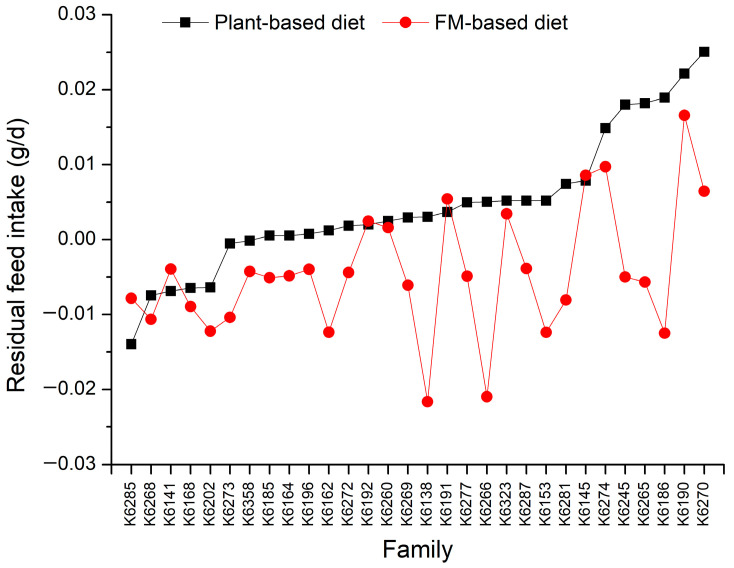
The variation in residual feed intake for 30 families under the plant-based and FM-based diet treatments.

The average RFI under the plant-based diet treatment (0.0049 g/d) was significantly higher than that under the FM-based diet treatment (−0.0045 g/d) (*p* < 0.001), indicating greater feed efficiency on the FM-based diet. The RFI values for 30 families under the two different diet treatments are depicted in Figure 1, showing a range of −0.014 to 0.025 g/d for the plant-based diet and −0.022 to 0.017 g/d for the FM-based diet, highlighting substantial inter-family variation. Furthermore, there were clear differences in RFI values among families between the two diets, with most families exhibiting lower utilization efficiency under the plant-based diet treatment compared to the FM-based diet treatment, but five families exhibited a utilization efficiency for the plant-based diet that was comparable to, or even better than, that for the FM-based diet.

### 3.2. Descriptive Statistics of Other Traits

**Figure 2:**
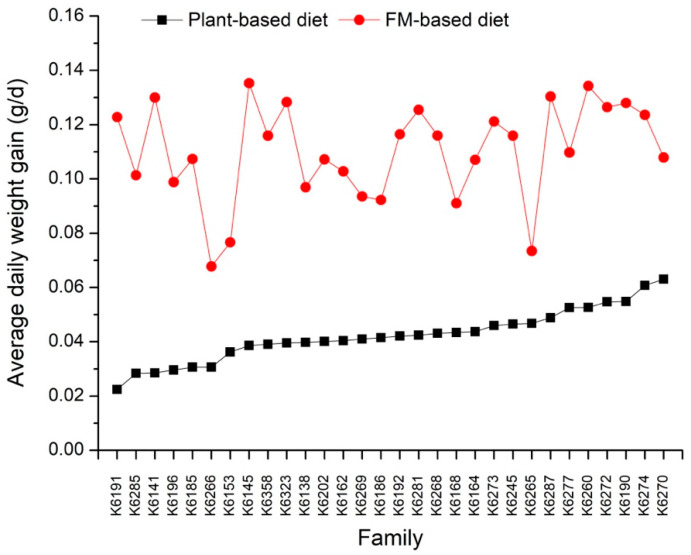
The variation in average daily weight gain for 30 families under the plant-based and FM-based diet treatments.

**Figure 3:**
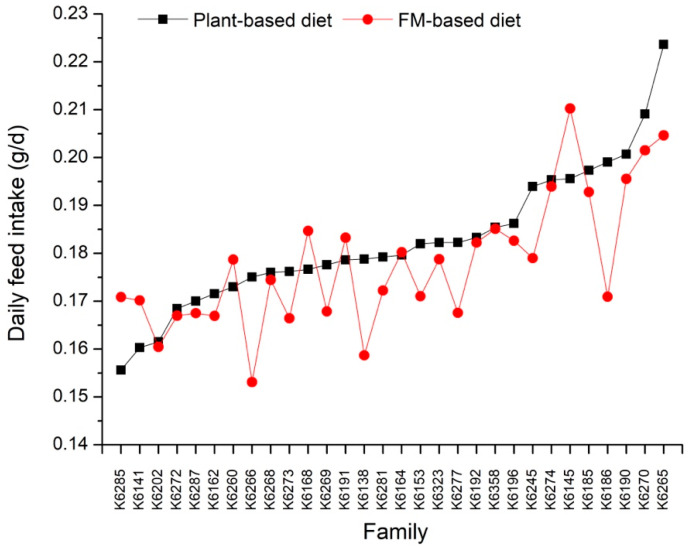
The variation in daily feed intake for 30 families under the plant-based and FM-based diet treatments.

Table 2 provides the simple means, minimum and maximum values, standard deviation, and coefficients of variation for FBW, ADG, and DFI under both the plant-based and FM-based diet treatments. Shrimp fed the FM-based diet showed significantly higher average FBW and ADG (*p* < 0.001) than those fed the plant-based diet. Additionally, greater inter-individual variations in FBW and ADG were observed under the plant-based diet treatment compared to the FM-based diet treatment, as indicated by the coefficients of variation (CV). All families exhibited noticeably higher ADG under the plant-based diet treatment compared to the FM-based diet treatment (Figure 2). In contrast, the average DFI of shrimp between the plant-based and FM-based diets was relatively similar. The variation in DFI among families did not differ noticeably between diets (Figure 3).

### 3.3. Variance Components and Heritability

Estimates of variance components and heritability for RFI, ADG, and DFI using bivariate analysis are given in Table 3. Under the plant-based diet treatment, RFI exhibited a high level of heritability (0.743 ± 0.157), as did DFI (>0.6) under the two diet treatments. However, there were no significant differences in heritability between diets (*p* > 0.05) for any trait recorded on both diets. Additionally, under the plant-based diet treatment, heritability estimates of RFI and DFI were significantly higher than that of ADG (*p* < 0.05), whereas under the FM-based diet treatment, there were no significant differences between these traits (*p* > 0.05).

### 3.4. Correlations and Genotype × Diet Interaction

Table 4 shows the genetic and phenotypic correlations between traits under the plant-based and FM-based diet treatments. RFI demonstrated high positive genetic correlations with ADG under both diet treatments (0.864 ± 0.112 and 0.586 ± 0.184), with no significant differences between diets (*p* > 0.05). However, the phenotypic correlations between RFI and ADG under both diet treatments were very weak. DFI displayed strong positive genetic correlations (>0.8) with RFI and ADG, which were not significantly different between diets (*p* > 0.05). Additionally, phenotypic correlations between DFI and ADG were significantly lower than their genetic correlations under both diet treatments (*p* < 0.01).

As shown in Table 5, genetic correlations between diets for RFI and DFI were high (0.646 ± 0.162 and 0.549 ± 0.163), indicating the existence of genotype × diet interactions for both traits. Conversely, the genetic correlation between ADG recorded on two diets was low (0.296 ± 0.259), showing no statistical differences from zero (*p* > 0.05), indicative of severe genotype × diet interaction. For any trait, the phenotypic correlation between diets was not statistically different from the genetic correlation between diets.

## 4. Discussion

### 4.1. The Effect of FM Levels on Shrimp Under Plant Protein Substitution

SBM and PM are commonly used as plant protein sources to replace FM in commercial shrimp diets in China. This study explored the feasibility of a fishmeal-free diet for shrimp using SBM and PM as substitutes. The FM-based diet resulted in a significant growth increase of 155.8%, but a slight decrease in DFI by 2.8% compared to the plant-based diet. Similar FI between diets demonstrated that shrimp readily accepted the plant-based diet, indicating no negative impact on diet palatability. Using SBM and PM as substitutes for FM, Dai et al. [37] found that whiteleg shrimp fed a diet with 25% FM showed a 7.06% increase in body weight compared to those on a 9% FM diet over a 75-day trial. Yue et al. [38] observed that a 10% FM diet resulted in a 5.7% weight reduction compared to a 20% FM diet, but a 13.3% increase compared to a 5% FM diet. These results indicate that a 10% FM content or higher does not significantly impact shrimp growth, while lower levels of FM in the diet lead to reduced weight gain. Additionally, our observations revealed that shrimp fed with plant-based diet had significantly higher RFI of 0.0049 g/d compared to the FM-based group’s −0.0045 g/d (*p* < 0.001), indicating that shrimp fed with higher levels of plant protein exhibited decreased feed efficiency. 

### 4.2. Factors Influencing the Calculation of RFI

In different animals, RFI is typically obtained by a multiple regression of feed intake on measures of various components, such as production, body weight gain, and body composition [39]. For example, the regression model for Japanese quails includes mean metabolic weight, weight gain, and egg mass production [40], while the model for French sheep includes liveweight gain, adjusted liveweight, back fat, and muscle depth [41]. Considering that feed consumption is primarily used for growth and maintenance during the growth stage of shrimp, in this study, metabolic mid-weight and ADG were incorporated into the model for shrimp as predictors of maintenance requirement and growth, respectively. 

It is crucial to determine the exponent of metabolic mid-weight. Most studies use a fixed value for the exponent of metabolic mid-weight to calculate expected feed intake, such as 0.75 for cattle [42], ram [43], and poultry [44,45], 0.8 for fish [46], and 0.6 for Pacific abalone [28]. Due to the lack of a reference for determining the exponent of metabolic body weight of shrimp, the exponent was treated as an unknown variable in the model in this study. The exponent of mid-weight estimated in the model for the plant-based diet (0.238) was lower than that for the FM-based diet (0.313), which may be attributed to different digestive capacities and metabolic utilization of nutrients for FM and plant protein. However, both exponents were lower than those (0.454–0.526) reported in a previous study [19]. It suggests that feed efficiency, when measured over an extended period, may vary depending on the developmental stages, rearing environment conditions, and management practices. Another point to note is that, judging from the variable coefficients of the regression model in this study, the metabolic mid-weight emerges as a more significant component for predicting feed intake than ADG, especially under a plant-based diet.

### 4.3. Analysis of the Heritability of RFI and Related Traits

The estimated heritability values for RFI were 0.743 ± 0.157 and 0.440 ± 0.125 under the plant-based and FM-based diet treatments, respectively, showing no significant difference between the two estimates. The lack of distinction in these values is primarily attributed to the larger standard errors caused by the small number of individuals within each family. These estimates align with previous heritability values (ranging from 0.580 to 0.747) derived from models without common environmental effects under a 25% FM diet [19,47]. However, when common environmental effects were accounted for, the heritability estimates ranged from 0.308 to 0.324 [47]. In comparison, studies investigating the heritability of RFI in terrestrial animals, such as pigs, cattle, and chicken, have reported moderate–high estimates (ranging from 0.10 to 0.47) [21,22,23,48,49]. Furthermore, the choice of different breeds can impact estimation of heritability of RFI, as highlighted by a review of a range of beef cattle breeds (0.16–0.43) [50]. Since this study’s design lacked half-sib families and featured relatively weak genetic relatedness among families, the common environmental effects resulting from the independent rearing of families before the experiment were difficult to distinguish, potentially leading to an overestimation of heritability. 

The heritability estimates of ADG under the plant-based and FM-based diet treatments were 0.314 ± 0.121 and 0.444 ± 0.126, respectively, which are lower than those reported (0.598 and 0.638) under a 25% FM diet [19]. The heritability estimate of DFI was very high (0.947 ± 0.158) under the plant-based diet treatment, appearing to be significantly inflated, while the FM-based diet estimate (0.678 ± 0.147) aligns with previous findings (0.664 and 0.696) under a 25% FM diet [19]. These estimates exceed reported heritability values for feed intake in fish (0.112 to 0.45) [20,51,52,53]. Considering the FE and growth performance of families under both diets, it is suggested that FE under plant-based diets has greater selection potential than growth.

### 4.4. Analysis of Genetic Correlations Between RFI and Other Traits

In livestock species, it is widely reported that there is a lack of genetic correlation between RFI and body weight gain [54,55,56,57]. Estimations of genetic correlation between RFI and weight gain are scarce in aquatic animals. Previous studies in shrimp [19] and Pacific abalone [28] also reported very low genetic correlations between RFI and ADG. In this study, while nearly no phenotypic correlations between RFI and ADG (0.033–0.103) was observed under both diets, the genetic correlations between RFI and ADG were high for both the plant-based (0.864 ± 0.112) and FM-based diets (0.586 ± 0.184). Silverstein et al. [58] found that, while under an apparent satiation feeding regime, there was a weak correlation between weight gain and RFI (−0.31) in rainbow trout. However, when fed a limited ration, this correlation became stronger, reaching −0.57. In our feeding trial, shrimp were actually fed a limited ration, which may contribute to the high genetic correlations between RFI and ADG. Additionally, this result suggests that the shrimp selected for greater growth consume more feed than needed for growth. The genetic correlations of RFI with DFI were also notably high under both the plant-based diet (0.992 ± 0.006) and the FM-based diet (0.930 ± 0.037), consistent with previous findings (0.815 ± 0.169 and 0.868 ± 0.098) on a 25% FM diet [19]. This result indicates that, under any type of diet, selecting for low RFI could improve feed efficiency and lead to a correlated reduction in feed intake based on the estimated genetic correlations. 

### 4.5. Analysis of Genetic Correlations Between Diets for RFI and Growth

Eknath et al. [59] suggested that a smaller genetic correlation between traits across different environments indicates a larger genotype by environment interaction effect. Considering diet as an environmental factor, the genetic correlation between RFI under the plant-based and FM-based diet treatments was 0.646 ± 0.162, indicating a moderate genotype × diet interaction effect. This suggests that the presence of FM in the diet impacts the feed utilization efficiency of whiteleg shrimp, with notable differences in FM requirement among different genotypes. More importantly, we found that five families exhibited a utilization efficiency for plant protein feed that was not lower than that for fishmeal feed. This reflects, from another perspective, the feasibility of conducting breeding programs aimed at improving the utilization efficiency of plant-based diet. 

Additionally, the study highlighted that ADG is notably influenced by the genotype × diet interaction, with a low genetic correlation of 0.296 ± 0.259 under two diet treatments. Dai et al. [37] found a high genetic correlation of 0.928 between body weight of whiteleg shrimp under a 9% FM diet and a 25% FM diet, suggesting no apparent genotype × diet interaction effect. In fish, Pierce et al. [15] observed a significant genotype × diet interaction effect (genetic correlation = 0.73 ± 0.13) for body weight in rainbow trout (*O. mykiss*) reared on plant-based and FM-based diets. Le Boucher et al. [14] similarly reported significant genotype × diet interaction for body weight in European sea bass (*Dicentrarchus labrax*), with genetic correlations ranging between 0.51 and 0.87, across all-plant-based and FM-based diets. However, Quinton et al. [51] found no significant genotype × diet interaction effect (genetic correlation > 0.9) for daily weight gain and feed efficiency in European whitefish (*Coregonus lavaretus* L.) fed on whole-FM diets versus low-FM–high-SBM diets. These findings suggest that the genotype × diet interaction effect on growth traits, caused by the presence of FM in the diet, is more significant than the interaction effect due to variation in FM content.

## 5. Conclusions

The significantly lower feed efficiency (FE) and growth of whiteleg shrimp on the plant-based diet compared to the FM-based diet suggest that a plant-based diet may lead to reduced yields, potentially affecting the profitability of shrimp culture. However, the estimated heritability of RFI under the plant-based diet reached 0.743 ± 0.157, indicating some potential for selective breeding, even though there may be an overestimation due to unaccounted-for common environmental effects. Notably, several families exhibited comparable or better FE under the plant-based diet than under the FM-based diet. Additionally, the presence of a moderate genotype × diet interaction for RFI between the plant-based and FM-based diets underscores the importance of considering selective breeding for improved feed efficiency in a plant-based diet environment.

## Figures and Tables

**Table 1:** Formulations and composition of the plant-based and FM-based diets.

Ingredients (g/kg)	Diet	
	FM-Based Diet	Plant-Based Diet
Fishmeal	250	0
Yeast	50	50
Peanut meal	90	200
Soybean meal	110	350
Wheat gluten	322	215
Soy phospholipid	18	25
Choline	5	5
Fish oil	30	30
Shrimp shell meal	100	100
Ca(H_2_PO_4_)_2_	15	15
Vitamin premix ^a^	5	5
Mineral premix ^b^	5	5
Proximate composition (%)		
Crude protein	38	38
Crude lipid	7.5	7.5
Crude fiber	3	3.3
Ash	5.3	6.6

^a^ Vitamin premix (mg or g/kg diet): thiamin, 25 mg; riboflavin, 45 mg; pyridoxine HCl, 20 mg; vitamin B_12_, 0.1 mg; vitamin K_3_, 10 mg; inositol, 800 mg; pantothenic acid, 60 mg; niacin, 200 mg; folic acid, 20 mg; biotin, 1.2 mg; retinol acetate, 32 mg; cholecalciferol, 5 mg; alpha-tocopherol, 120 mg; wheat middling, 3.67 g. ^b^ Mineral premix (mg or g/kg diet): MgSO_4_·7H_2_O, 1200 mg; CuSO_4_·5H_2_O, 10 mg; ZnSO_4_·H_2_O, 50 mg; FeSO_4_·H_2_O, 80 mg; MnSO_4_·H_2_O, 45 mg; CoCl_2_·6H_2_O (1%), 50 mg; NaSeSO_3_·5H_2_O (1%), 20 mg; Ca(IO_3_)_2_·6H_2_O (1%), 60 mg; zoelite, 3.485 g.

**Table 2:** Descriptive statistics of FBW, ADG, and DFI under the plant-based and FM-based diet treatments, respectively.

Trait	Diet	Mean	Max	Min	SD	CV (%)
FBW (g)	Plant-based	5.773	12.550	2.280	1.631	28.252
	FM-based	8.643	19.620	3.610	2.118	24.505
ADG (g/d)	Plant-based	0.043	0.187	0.005	0.025	58.140
	FM-based	0.110	0.250	0.009	0.034	30.909
DFI (g/d)	Plant-based	0.183	0.255	0.119	0.019	10.383
	FM-based	0.178	0.246	0.114	0.021	11.798

Note: FBW, the body weight at the end of the experiment; ADG, average daily weight gain; DFI, daily feed intake; SD, standard deviation; CV, coefficients of variation.

**Table 3:** Variance components and heritability estimates for RFI, ADG, and DFI under the plant-based and FM-based diet treatments.

Trait	Diet	Variance Components			$h^{2}$ ± s.e.
		$\sigma_{a}^{2}$	$\sigma_{e}^{2}$	$\sigma_{p}^{2}$	
RFI (g/d)	Plant-based	1.80 × 10^−4^	6.20 × 10^−5^	2.42 × 10^−4^	0.743 ± 0.157
	FM-based	7.96 × 10^−5^	1.01 × 10^−4^	1.81 × 10^−4^	0.440 ± 0.125
ADG (g/d)	Plant-based	1.96 × 10^−4^	4.28 × 10^−4^	6.24 × 10^−4^	0.314 ± 0.121
	FM-based	5.20 × 10^−4^	6.50 × 10^−4^	1.17 × 10^−3^	0.444 ± 0.126
DFI (g/d)	Plant-based	2.78 × 10^−4^	1.55 × 10^−5^	2.93 × 10^−4^	0.947 ± 0.158
	FM-based	1.77 × 10^−4^	8.45 × 10^−5^	2.62 × 10^−4^	0.678 ± 0.147

Note: RFI, residual feed intake; ADG, average daily weight gain; DFI, daily feed intake.

**Table 4:** Genetic and phenotypic correlations between traits under a single diet treatment.

Correlation	Diet	Trait		
		RFI-ADG	RFI-DFI	ADG-DFI
Genetic	Plant-based	0.864 ± 0.112	0.992 ± 0.006	0.900 ± 0.078
	FM-based	0.586 ± 0.184	0.930 ± 0.037	0.827 ± 0.089
Phenotypic	Plant-based	0.103 ± 0.079	0.942 ± 0.009	0.407 ± 0.062
	FM-based	0.033 ± 0.070	0.859 ± 0.017	0.518 ± 0.050

Note: RFI, residual feed intake; ADG, average daily weight gain; DFI, daily feed intake.

**Table 5:** Genetic and phenotypic correlations between the plant-based and FM-based diets for RFI, ADG, and DFI, respectively.

Correlation Between Diets	Trait		
	RFI	ADG	DFI
Genetic	0.646 ± 0.162	0.296 ± 0.259	0.549 ± 0.163
Phenotypic	0.369 ± 0.132	0.110 ± 0.102	0.440 ± 0.164

Note: RFI, residual feed intake; ADG, average daily weight gain; DFI, daily feed intake.

## Data Availability

The original contributions presented in the study are included in the article, further inquiries can be directed to the corresponding author.
